# Effects of traditional Chinese mind–body exercises on older adults with cognitive impairment: A systematic review and meta-analysis

**DOI:** 10.3389/fneur.2023.1086417

**Published:** 2023-04-03

**Authors:** Ke-ru Yao, Qin Luo, Xi Tang, Zhi-han Wang, Lu Li, Lu Zhao, Li Zhou, Ling Li, Li Huang, Xin-hong Yin

**Affiliations:** ^1^School of Nursing, University of South China, Hengyan, China; ^2^Second Affiliated Hospital of University of South China, Hengyang, Hunan, China

**Keywords:** cognitive impairment, elderly, mind–body exercise, Baduanjin, Tai Chi

## Abstract

**Objective:**

To determine the effectiveness of traditional Chinese mind–body exercises in improving cognition, memory, and executive function in older adults with cognitive impairment.

**Data sources:**

Relevant English and Chinese language studies published until September 14th, 2022 were retrieved from PubMed, Web of Science, Cochrane Library, Embase, CINAHL, WAN FANG DATA, VIP Information, CNKI, and SinoMed databases.

**Review methods:**

Randomized controlled trials assessing traditional Chinese mind–body exercises (Tai Chi, Baduanjin, Qigong, Mind–Body Therapies, and Yijinjing) in older adults with cognitive impairment were included. Two researchers independently identified eligible studies and extracted data. A risk-of-bias assessment was performed using the Cochrane Risk of Bias Tool.

**Results:**

This study included 15 randomized controlled trials (1,127 participants) from China, Thailand and American. Most studies had a high risk of bias in the blinding of participants and researchers, one study had a high risk of bias in the random sequence generation and two studies had a high risk of bias in the incomplete outcome data. Compared with conventional therapy alone, traditional Chinese mind–body exercises significantly improved global cognitive function (*p* < 0.00001), and Baduanjin could improve the global cognitive function (*p* < 0.00001), memory function (*p* < 0.0001), and executive function (*p* < 0.0001) outcomes after treatment, and significantly improved some dimensional scores on the auditory verbal learning test after treatment (*p* = 0.04).

**Conclusion:**

Compared with conventional therapy, traditional Chinese mind–body exercises (Tai Chi, Baduanjin, and Qigong) significantly improved global cognitive function, and Baduanjin could improve global cognitive function, memory function, and executive function in older adults with cognitive impairment.

**Systematic Review Registration:**

https://www.crd.york.ac.uk/PROSPERO/#searchadvanced, CRD42022327563.

## Introduction

With increasing longevity, cognitive impairment that affects primarily older adults has become a major public health concern (1). People living with dementia suffer from progressive cognitive impairment. Alzheimer’s disease (AD) is the most common type of dementia, constituting about 90% of the cases of dementia in this population (2). New diagnostic criteria for AD, which clearly stated that AD is a continuous pathophysiological process, including subjective memory complaints, mild cognitive impairment (MCI) and Alzheimer’s disease (3). However, there is no cure for dementia, targeted intervention is an effective starting point to prevent and delay the occurrence of dementia.

Studies show that physical exercise interventions are effective in maintaining or improving cognitive function in older adults (4). At present, exercise intervention for patients with cognitive impairment mainly focuses on aerobic exercise, resistance training, endurance training, and so on (5). However, for the elderly, with the increase of age and the decline of physical function, they often cannot bear and adhere to high-intensity sports training for a long time. Traditional Chinese mind–body exercises (TCEs), which consist of gentle movements, breathing techniques, and meditation (6), are deeply loved by the elderly. The National Fitness Guide issued by the State Sports General Administration of China in 2017 pointed out that TCEs, which include Tai Chi, Baduanjin, Qigong, Yijinjing, and Wuqinxi, are gentle and safe for older adults, with an emphasis on the combination of meditation and physical activities (7). Meanwhile, an increasing number of studies have reported positive effects of traditional Chinese mind–body exercises on cognitive ability in different populations (8, 9). Tai Chi is a mind–body exercise that was originally developed as a martial art in China. As a multicomponent intervention, Tai Chi combines coordination of slow movements with mental focus, deep breathing, and relaxation (10). Qigong involves the performance of a static or dynamic set of meditative exercises with the intention of coordinating one’s mental energy, breathing, and physical movement (11). Baduanjin is a traditional Chinese qigong exercise that consists of eight movements. It is a safe aerobic exercise based on the common rules of qigong (12). The Yijinjing exercise consists of linear movements and requires isolated joint movements that emphasize the combination of symmetrical physical postures, meditative mind, and breathing techniques in a harmonious manner (13). The Wuqinxi exercise routine was originally choreographed by an ancient Chinese physician in the Donghan Dynasty, that imitates the movements of tiger, deer, bear, ape, and bird, focuses on the combination of dynamic and static or inside and outside(14). The common characteristics of traditional Chinese exercises (TCEs) include that they are slow, relaxing, and systematic, making suitable for physically weak patients. Indeed, TCEs not only have aerobic effects, which are an important mediator to promote cognitive fitness, but also have beneficial effects on reversing negative mood among older adults, which is a prominent risk factor for cognitive decline (8). In recent years, an increasing number of clinical studies have examined the use of TCEs in patients with cognitive impairment and a growing body of systematic reviews (15, 16) has demonstrated that TCEs can effectively improve the cognitive function of the elderly, however, these reviews had several limitations, one of which is that several recent studies had been recently published, thus, existing evidence must be synthesized and updated. Another is that this review only focused on Tai Chi, Baduanjin and Qigong three types, older adults who practice other TCEs, such as Yijinjing and Wuqinxi, were not included in this review. Therefore, findings cannot be applied to a broader population. In addition, we performed meta regression analysis on the duration of total treatment and intensity of the TCEs varied among cognitive functions, therefore, the present review evaluated existing studies on this topic to address these gaps in research.

The objectives of this review were to examine the effects of different TCEs on the cognitive function of older adults with cognitive impairment.

## Methods

### Search strategy

We searched five English databases, namely, PubMed, Web of Science, The Cochrane Library, Embase, and CINAHL, and four Chinese databases, namely, the National Knowledge Infrastructure (CNKI), W ANFANG DA TA, VIP Information, and SinoMed. Relevant randomized controlled trials with the MeSH term “Aged, Tai Ji, Mind–Body Therapies, Mindfulness, Qigong, Cognitive Dysfunction” were searched for; studies published in English or Chinese from the databases’ inception until September 14th, 2022 were searched. The search strategies have been provided as examples in the appendix.

### Inclusion and exclusion criteria

Studies were included in this meta-analysis if they met the following criteria: (1) studies with patients over the age of 60 with clinically diagnosed cognitive impairment; (2) studies in which control-group patients received conventional therapy, maintained their daily routine, and did not receive any other exercise therapy; (3) in the case of studies with more than two groups, only the TCEs (Tai Chi, Baduanjin exercise, Qigong, Mind–Body Therapies, Yijinjing) and non-physical (control) groups were chosen; (4) studies with at least one outcome measuring cognitive function, such as the Montreal Cognitive Assessment, Mini-Mental State Examination, and Wechsler Memory Scale, and (5) studies designed as randomized controlled trials.

The exclusion criteria were as follows: (1) the experimental group engaged in exercises other than TCEs (Tai Chi, Baduanjin exercise, Qigong, Mind–Body Therapies, Yijinjing); (2) documents in which studies or data were repeated; and (3) duplicate data. Case of duplicate reports, researchers either extracted data from each report separately and then combined the information from multiple data-collection forms, or they extracted the data from all the reports directly into a single data-collection form, as appropriate; (4) animal experiments.

### Data extraction and quality assessment

According to the retrieval strategy, the titles, authors, and abstracts of all related documents were imported into EndNote X9 software, and repetitive documents were eliminated. The deduplicated titles and abstracts were then read to eliminate documents that might meet the selection criteria. Finally, the documents that met the selection standards were screened using full-text reading. The literature was screened independently by two researchers, and in the case of differences, a third researcher was invited to discuss and resolve the problem.

The following information was extracted from the selected studies: general data (title, first author, publication date, location, etc.) and research characteristics (grouping methods, sample size, details of TCEs (Tai Chi, Baduanjin, Qigong, Mind–Body Therapies, Yijinjing) training such as frequency and duration, outcome indicators, etc.).

We used the Cochrane Handbook for Systematic Reviews of Interventions 5.1 to assess the risk of bias of the selected studies in the following categories: random allocation, concealed allocation, blinding of participants and personnel, detection bias, attrition bias, reporting bias, and others. The risk of bias was recorded as high, low, or unclear. Two researchers independently assessed all items, and disagreements were resolved by consensus in consultation with a third researcher.

Stata 16.0 software was used to test the publication bias of the literature included in the research results. If the number of studies included in the analysis was more than 4, the publication bias was evaluated by egger test (*p* < 0.05). If there is publication bias, its impact on the results is further evaluated by the clipping method. Publication bias was inspected for asymmetry using funnel plots.

### Statistical analysis

The Cochrane RevMan software (version 5.4; The Cochrane Collaboration/The Nordic Cochrane Center, Copenhagen, Denmark) was used to synthesize the outcomes. The primary outcome was cognitive function (Montreal Cognitive Assessment and Mini-Mental State Examination), secondary outcome included memory (the auditory verbal learning test) and executive function [Trail-Making Test (TMT)]. We performed a statistical analysis by using the weighted mean difference with 95% confidence interval. We used the random-effects model for meta-analysis when significant heterogeneity existed (*p* < 0.05, *I*^2^ > 50%) among the included studies. Otherwise, the fixed-effects model was applied.

## Results

**Figure 1:**
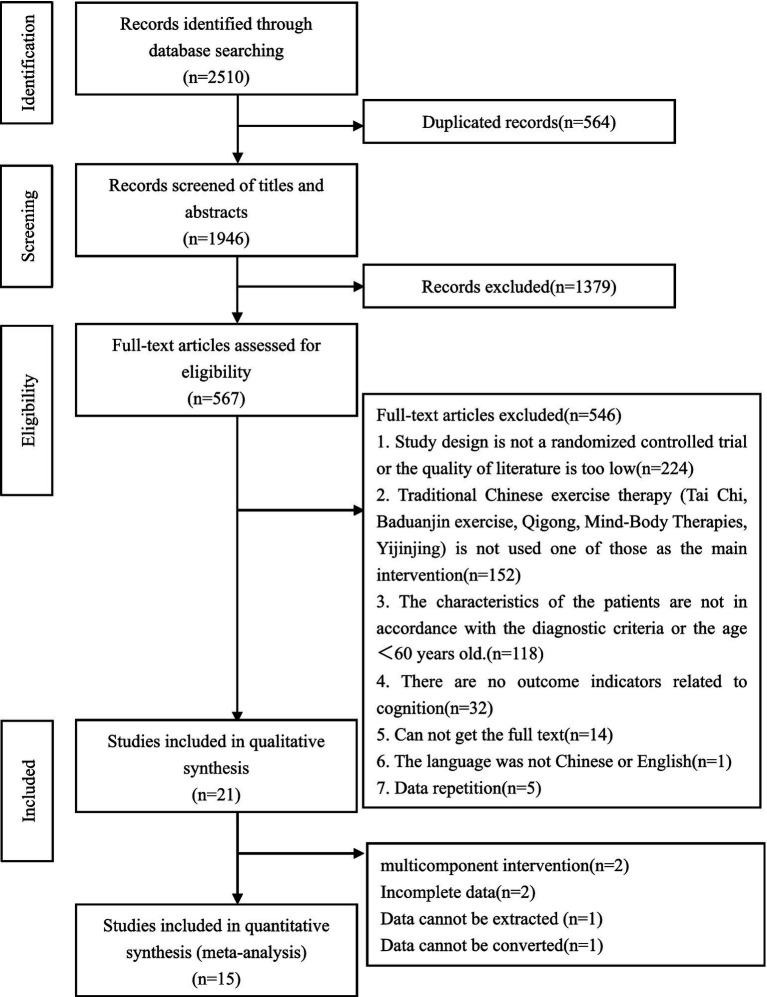
Flow chart of study selection.

The results of the search process are shown in Figure 1. A total of 2,510 studies of potential relevance were retrieved from the 9 databases. Of these, 21 randomized controlled trials were considered eligible for analysis. Six of these studies were not included in the meta-analysis. Two studies were multicomponent interventions (15, 16). Two studies’ data were incomplete (11, 17). (There was no mean or standard deviation in the Montreal Cognitive Assessment.) One study’s data cannot be extracted (18) (MMSE’s data). One study’s data cannot be converted (19) (TMT). Thus, finally, 15 randomized controlled trials with 1,127 participants were included in this meta-analysis. Their characteristics are summarized in Table 1. Of these studies, 13 were conducted between 2013 and 2022 in China, one was from Thailand and one was from the United States.

**Table 1:** Characteristics of the included studies.

Studies	Location (language)	Total sample size (control/experiment)	Intervention		Experimental program	Duration (mo)	Outcomes
			Control	Experiment			
22	Fuzhou, China (English)	40 (20/20)	Usual physical activity	Baduanjin	60 min/session, three times a week	6	MoCA-Chinese, WMS-CR, WMS-MQ
9	Nanjing, China (Chinese)	57 (28/29)	Routine treatment and health education	Routine treatment and health education + Baduanjin	50 min/session, three times a week	6	MoCA, RBMT-II, DSST, TMT
32	Da Qing, China (English)	60 (30/30)	Mindfulness	Mindfulness + Tai Chi Chuan	60 min/session, twice a week	3,6,12	MMSE, Frailty Criteria Rate
34	Harbin, China (English)	70 (35/35)	Gymnastics practice	Baduanjin	60 min/session, five times a week	3	SMCQ, AVLT, TMT
12	Fuzhou, China (Chinese)	102 (51/51)	Routine health education	Routine health education + Baduanjin	60 min/session, three times a week	6	MoCA, TMT, ROCF
35	Chang Sha, China (English)	92 (49/43)	Routine health education	Routine health education + Tai Chi Chuan	≥45 min/session, four times a week	4	AVLT, TMT
26	Fuzhou, China (English)	40 (20/20)	Routine health education	Routine health education + Baduanjin	60 min/session, three times a week	6	MoCA-BJ, ALFF
25	Tianjin, China (English)	40 (20/20)	Cognitive Training	Cognitive Training + Qigong	60 min/d	3	MoCA, LOTCA
23	Beijing, China (English)	74 (38/36)	Routine treatment and personalized daily care	Routine treatment and personalized daily care + Tai Chi Chuan	20 min/session, three times a week	5,10	MMSE, MoCA, WHO-UCLA-A VLT, TMT
36	Chiang Mai, Thailand (English)	56 (27/29)	Routine health education	Routine health education + Tai Chi Chuan	50 min/session, three times a week	6	LM-delayed recall, DS, Block Design, TMT(B-A)
28	Fuzhou, China (Chinese)	68 (32/36)	Routine health education	Routine health education + Baduanjin	60 min/session, three times a week	6	MoCA-BJ, WMS, DST, TMT, TAP
27	Beijing, China (Chinese)	85 (43/45)	No intervention	Qigong	30 min/session, twice/d, ≥5 days a week	6	MoCA, MMSE
33	Hong Kong, China (English)	261 (169/92)	Muscle- stretching and toning	Tai Chi Chuan	≥30 min/session, 3 days a week	12	MMSE, ADAS-Cog, Delay recall, Digit span, Visual span, CDR, Dementia
31	American (English)	46 (24/22)	Stretching Exercise	Tai Chi Quan	60 min/session, two times/week	4	MoCA,TMT-B, Digit span, Verbal Fluency
30	Taiwan, China (English)	36 (18/18)	Daily Physical Activities	Tai Chi Quan	50 min/session, three times a week	3	MoCA,TMT, CVLT, SWCT

**Figure 2:**
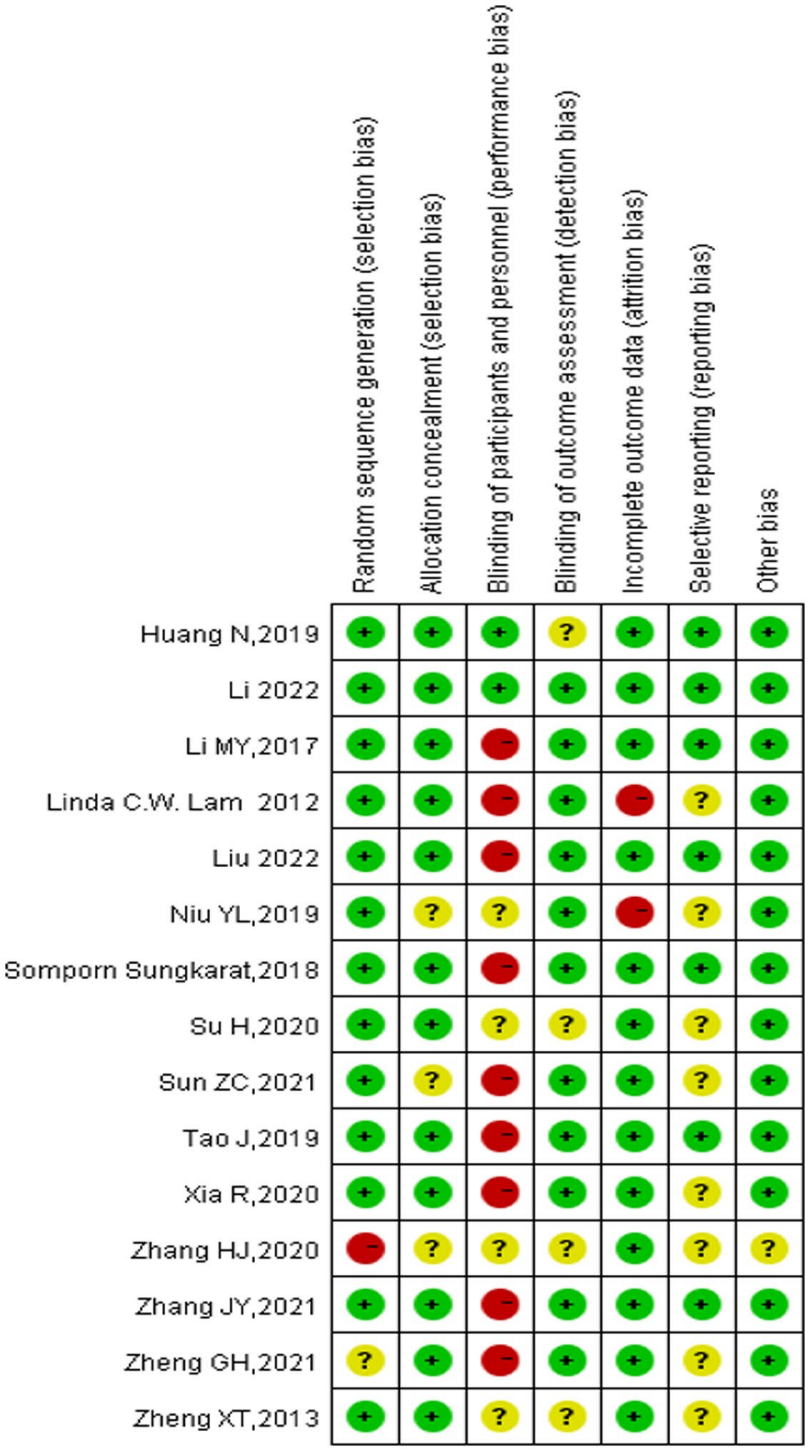
Risk-of-bias graph.

**Figure 3:**
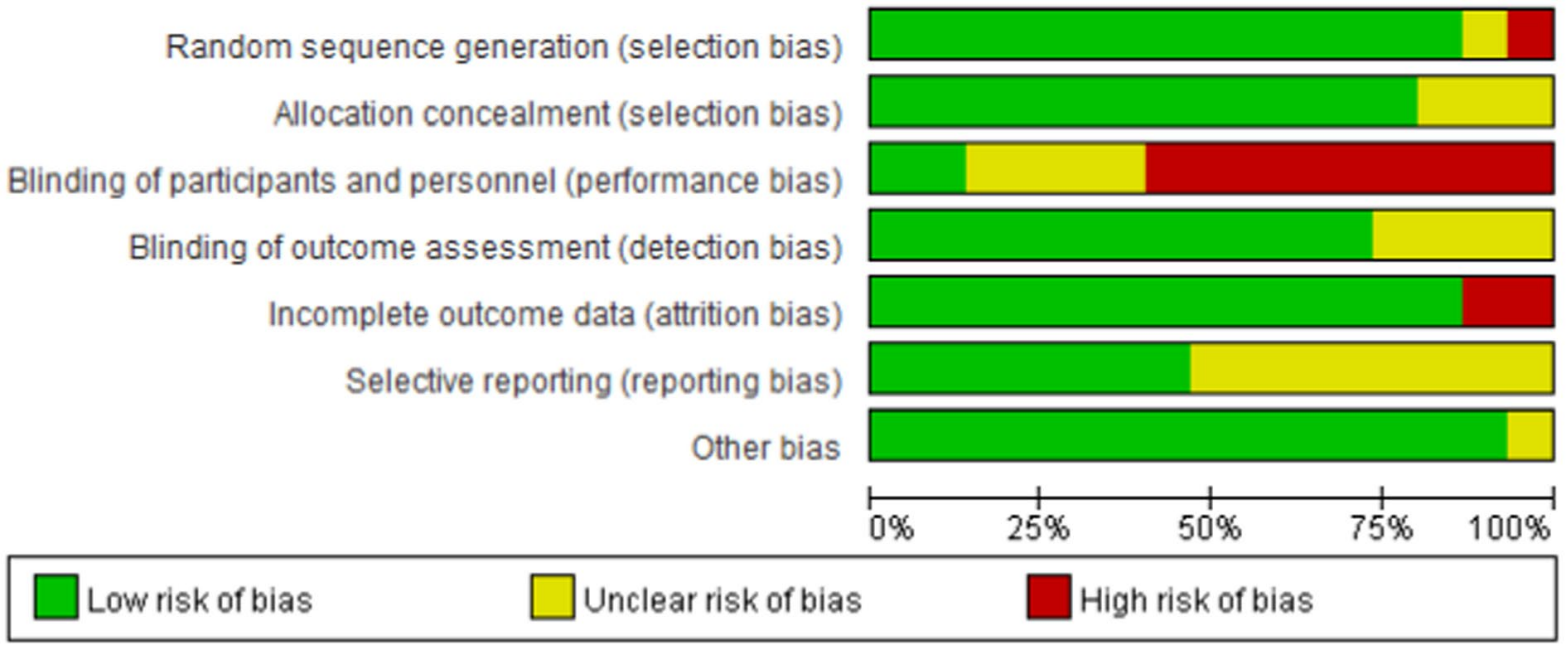
Risk-of-bias summary.

TCEs that were included in our study, were Tai Chi, Baduanjin, and Qigong. The specifics of the TCEs programs varied among the studies, with 20–60-min sessions per day, 3–7 sessions per week, and a total duration of 3–12 months. The results of the risk of bias assessments of each included study are shown in a risk-of-bias graph (Figure 2) and a risk-of-bias summary (Figure 3).

The cognitive function indices Montreal Cognitive Assessment and Mini-Mental State Examination were combined because both are screening tools for cognitive impairment (20).

The memory function was evaluated by the auditory verbal learning test (21, 22). Chinese-AVLT includes three subtests: immediate recall, short-term delayed recall, and long-term delayed recognition. The WHO-UCLA-AVLT includes immediate recall and delayed recall (23).

Executive function was assessed using the Trail-Making Test (TMT) Part B–A (24). The difference between the times taken to complete Parts A and B (B–A) was used to index task switching, a subdomain of executive function. In the TMT-A part, the subjects were asked to connect the numbers in the project from 1 to 25 in order from small to large, while TMT-B requires participants to draw a line alternating between numbers and letters in ascending order between the number and the letter. The scoring index is the amount of time taken, and the longer the time is, the worse the executive function is. The TMT-B/TMT-A is considered a valid index of executive ability.

### Primary outcome- global cognitive function

**Figure 4:**
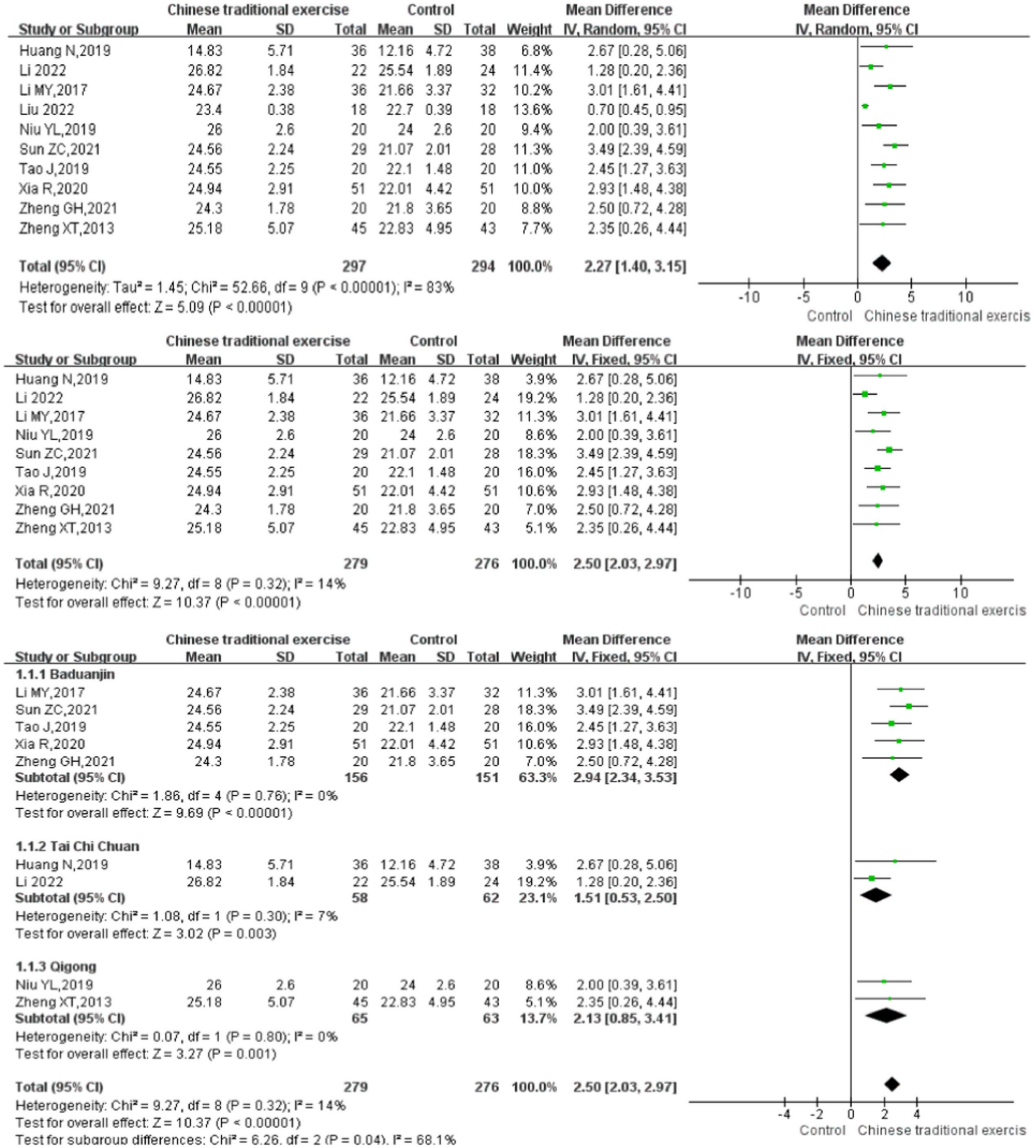
Total Montreal cognitive assessment scores after traditional Chinese exercises.

A total of 10 studies (9, 22, 23, 25–31) with 591 patients assessed the Montreal Cognitive Assessment scores after treatment. High significant heterogeneity was found among these studies (*I*^2^ = 83%), after heterogeneity tests we find that an article is a source of heterogeneity (30), heterogeneity was reduced after remove this article (*I*^2^ = 14%), then fixed-effects model was used. The results showed that the impact of TCEs on cognitive function (MoCA) in elderly with cognitive impairment is statistically significant [MD = 2.50, 95% CI (2.03, 2.97), *p* < 0.00001]. And meta-analysis showed that the curative effects of Baduanjin [MD = 2.94, 95% CI (2.34, 3.53), *p* < 0.00001], Tai Chi [MD = 1.51, 95% CI (0.53, 2.50), *p* = 0.003], and Qigong [MD = 2.13, 95% CI (0.85, 3.41), *p* = 0.001] were significantly better than those of the control group (Figure 4).

**Figure 5:**
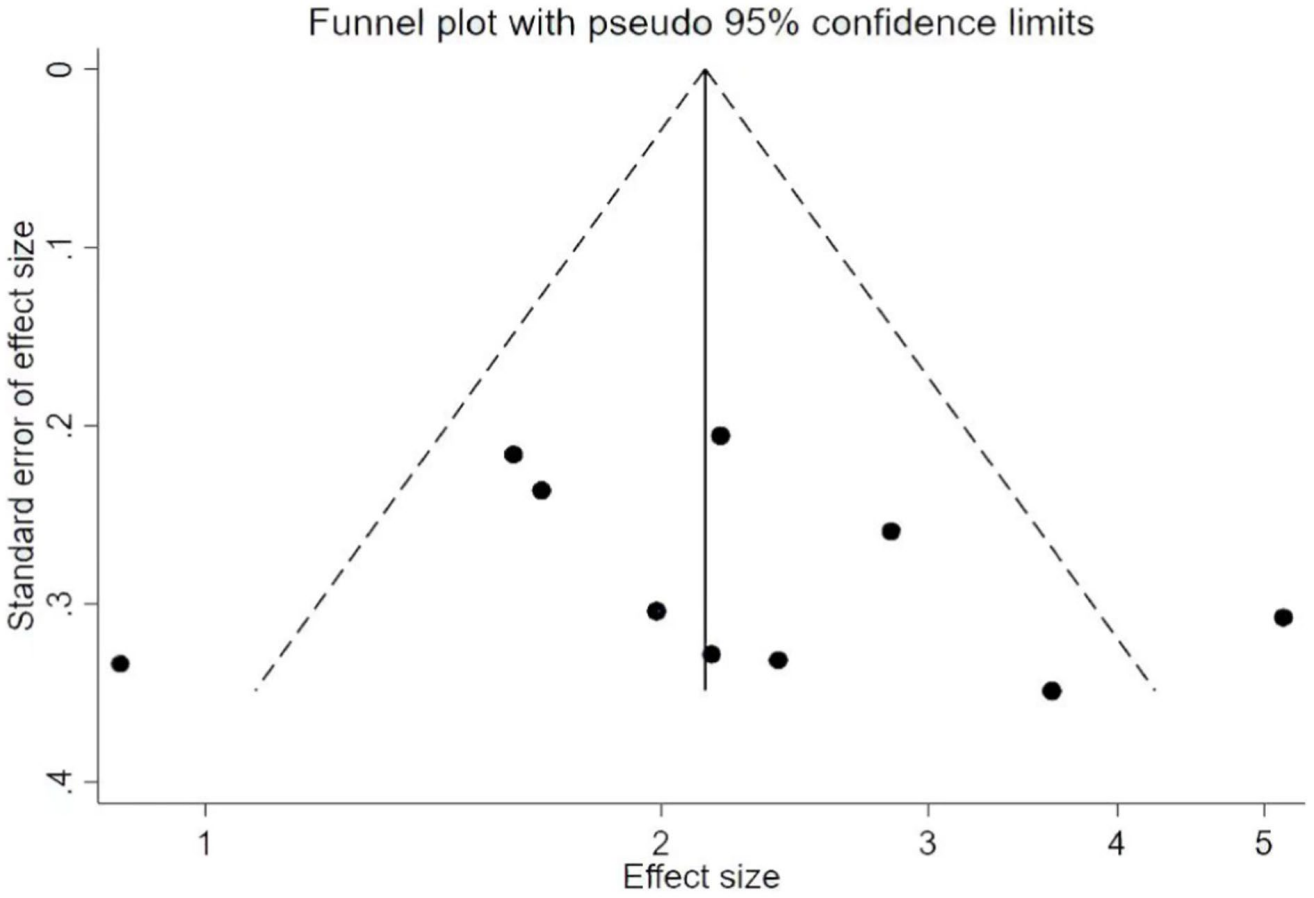
Publication bias of Montreal cognitive assessment scores.

The meta-regression analysis of the MoCA scores showed that there was no significance in intervention times [95% CI (−0.05, 0.05), *p* = 0.961], intervention duration [95% CI (−1.17, 1.34), *p* = 0.844] and single intervention time [95% CI (−0.21, 0.24), *p* = 0.754] (Table 2). And as for the control groups involving active procedures and year of publication, there is also no significance [95% CI (−4.33, 3.97), *p* = 0.869; 95% CI (−1.22, 1.16), *p* = 0.924] (Table 2). Publication bias of Montreal cognitive assessment scores can be seen in the funnel plot (Figure 5), Egger’s regression indicates that there is little possibility of publication bias in the included studies (*p* = 0.536).

**Table 2:** Meta-regression analysis of potential moderators MoCA to explain heterogeneity of older people with cognitive impairment.

	Meta-regression coefficient	95%CI	*P*
Year of publication	−0.029992	−1.221405 to 1.161421	0.924
Intervention times	−0.0007185	−0.0561566 to 0.0547196	0.961
Intervention duration	0.0642515	−1.175498 to 1.304001	0.844
Single intervention time	0.0188652	−0.2068968 to 0.2446272	0.754
Control groups involve active procedures	−0.1808187	−4.33422 to 3.972582	0.869

**Figure 6:**
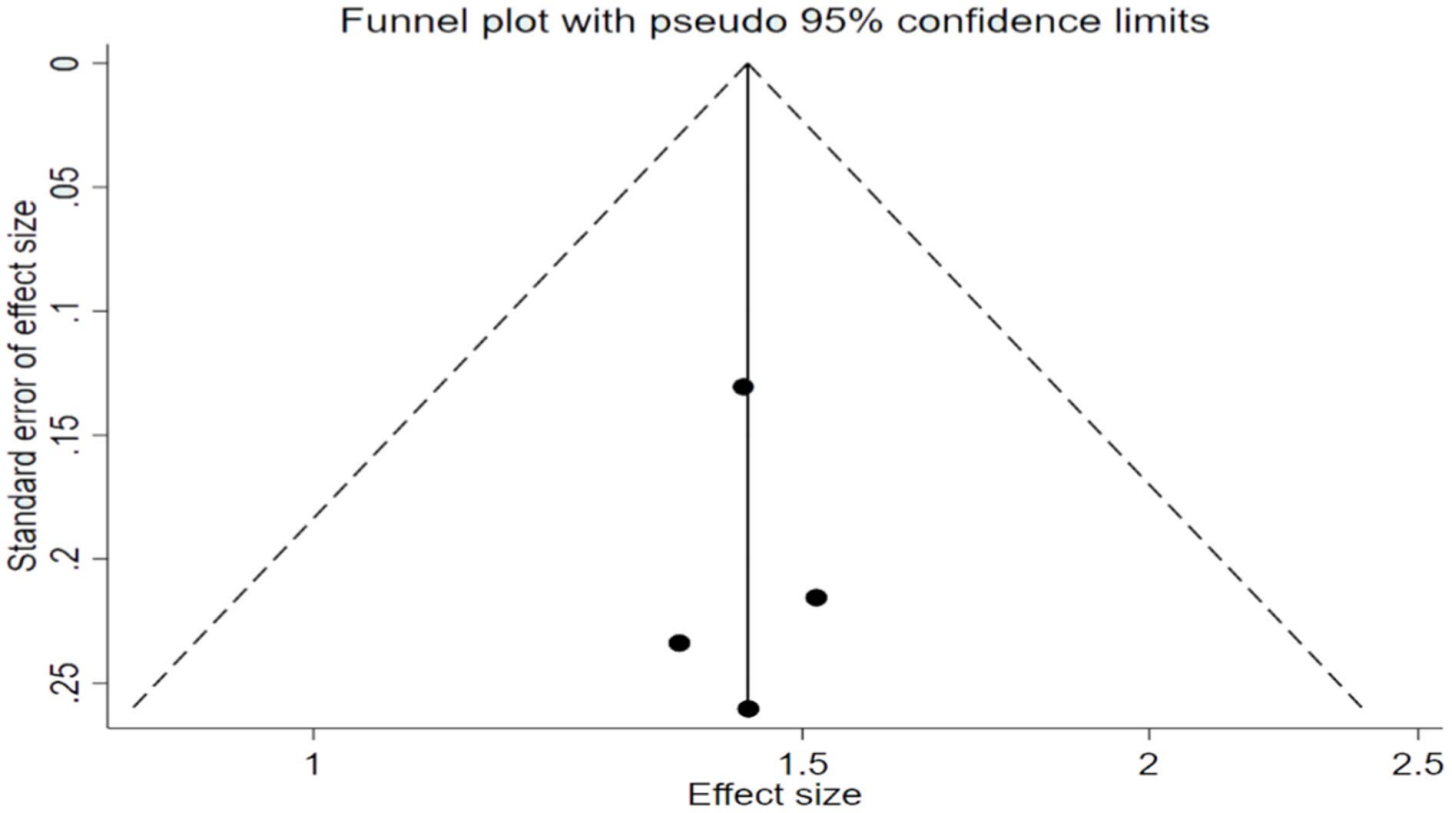
Mini-mental state examination scores after traditional Chinese exercises.

**Figure 7:**
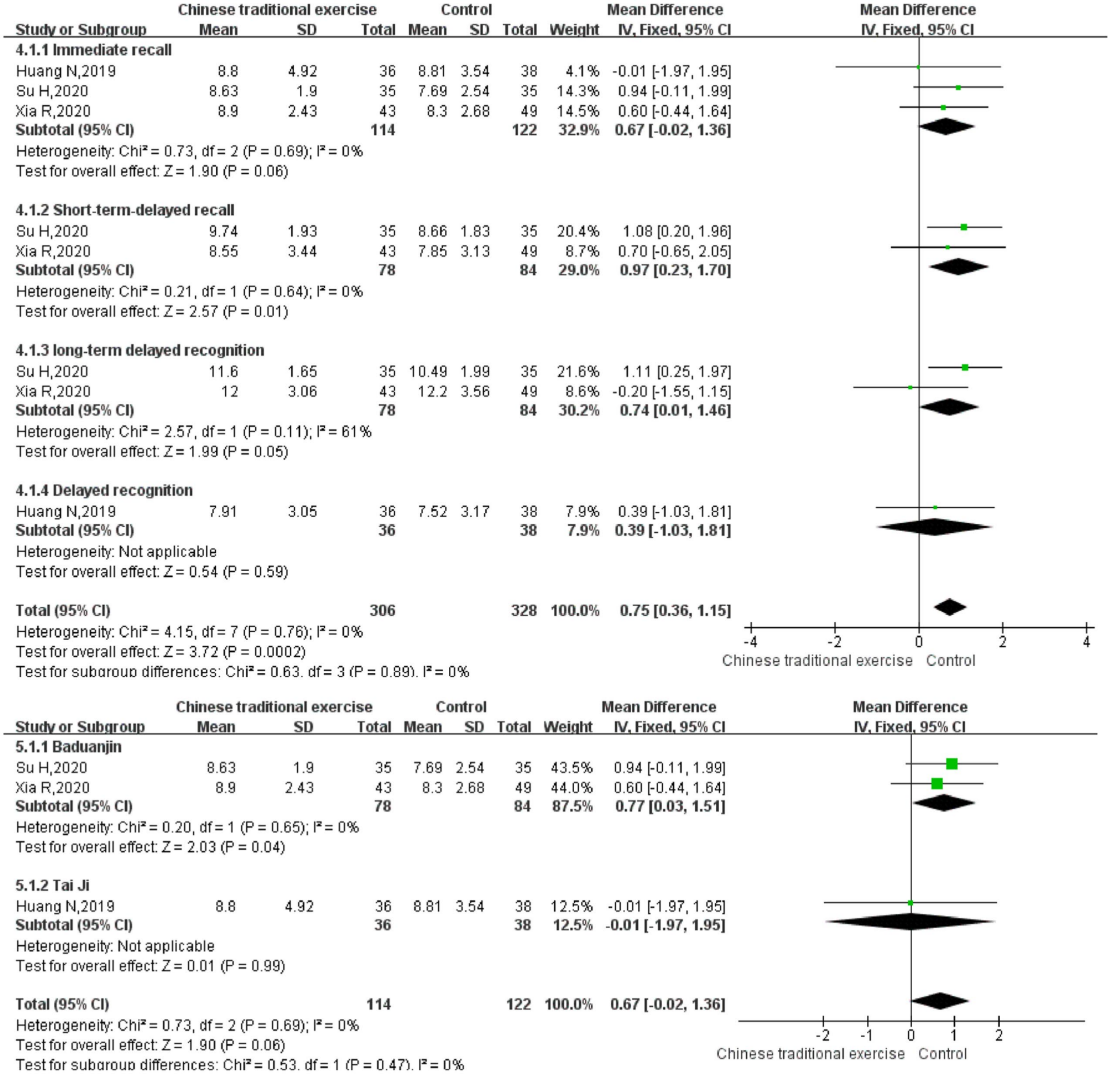
Publication bias of mini-mental state examination scores.

Four studies (25, 27, 32, 33) with a total of 483 participants assessed the Mini-Mental State Examination scores, which showed no heterogeneity between the studies (*p* > 0.05, *I*^2^ < 50%), hence, the fixed-effects model was used. The results indicated that TCEs training improved the Mini-Mental State Examination scores of older adults with cognitive impairment [MD = 1.06, 95% CI (0.51, 1.61), *p* = 0.0002] (Figure 6). Publication bias of Mini-Mental State Examination scores can be seen in the funnel plot (Figure 7), Egger’s regression indicates that there is little possibility of publication bias in the included studies (*p* = 0.991).

### Secondary outcome- memory and executive function

**Figure 8:**
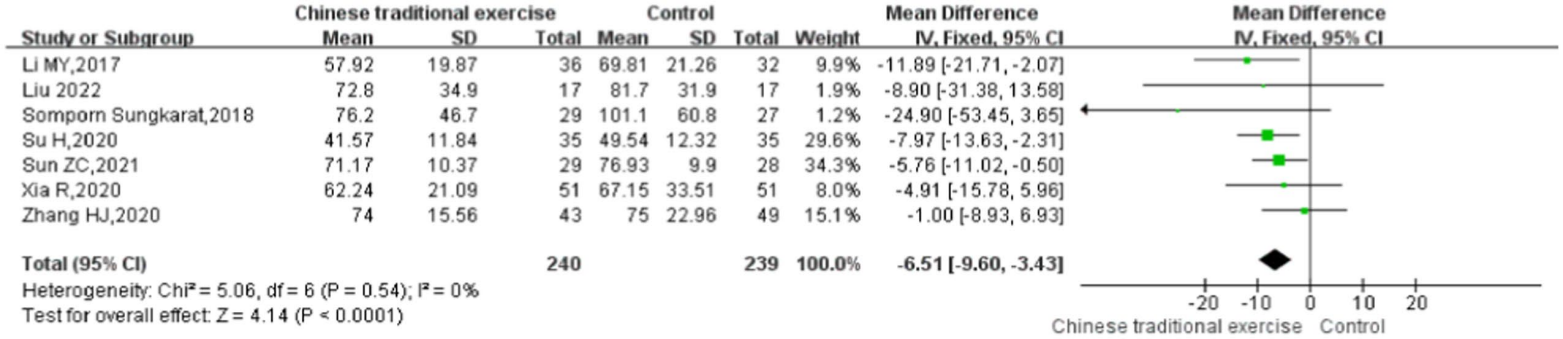
Auditory verbal learning test after traditional Chinese exercises.

AVLT includes three subtests: immediate recall, short-term delayed recall, and long-term delayed recall. Therefore, we further performed subgroup analysis. According to the meta-analysis, traditional Chinese mind–body exercises can improve short-term delayed recall [MD = 0.97, 95% CI (0.23, 1.70), *p* = 0.01]. However, there is no statistically significant difference in long-term delayed recall [MD = 0.74, 95% CI (0.01, 1.46), *p* = 0.05] and immediate recall [MD = 0.67, 95% CI (−0.02, 1.36), *p* = 0.06] (Figures 5–8). The AVLT-immediate recall was analyzed in 3 studies (25, 29, 34) with 236 participants. The meta-analysis showed that the AVLT-immediate recall of different TCEs was statistically different. It was shown that the curative effect of Baduanjin was better than that of conventional therapy [MD = 0.77, 95% CI (0.03, 1.51), *p* = 0.04], but there was no statistically significant difference between Tai Chi and conventional therapy in AVLT-immediate recall [MD = −0.01, 95% CI (−1.97, 1.95), *p* = 0.99] (Figures 7, 8). These results indicated that TCEs improved the memory function of older adult with cognitive impairment, especially the scores of short-term delayed recall.

**Figure 9:**
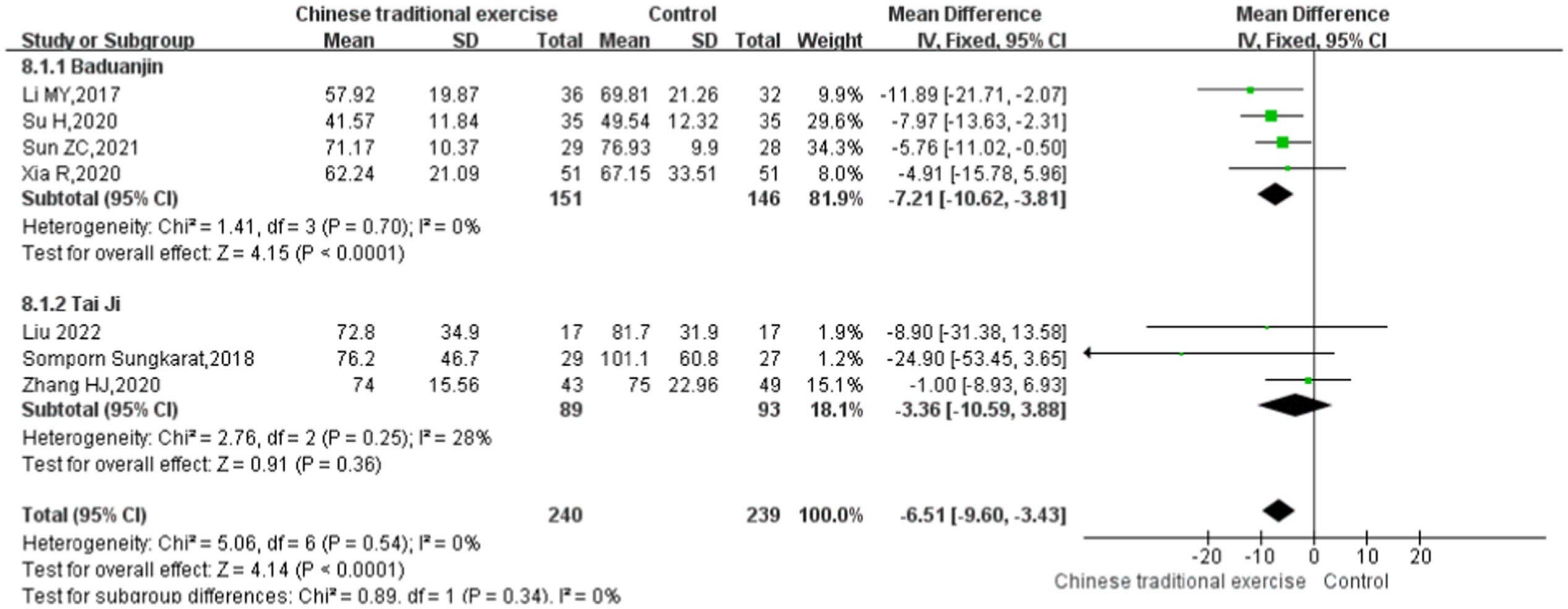
Trail-making test (TMT) after traditional Chinese exercises.

In this study, TMT was used to evaluate the executive function of the subjects (33). Seven studies (9, 28–30, 34, 36, 37) with 450 participants assessed the Trail-Making Test scores. No significant heterogeneity was found among these studies (*p* > 0.05, *I*^2^ < 50%), so the fixed-effects model was used. The results showed that the impact of TCEs on executive function (TMT) of elderly patients with cognitive impairment is statistically significant [MD = -6.51, 95% CI (−9.60, −3.43), *p* < 0.0001]. The meta-analysis showed that the scores of the Trail-Making Test for different TCEs were statistically different. It was shown that the curative effect of Baduanjin was better than that of conventional therapy [MD = –7.21, 95% CI (−10.62, −3.81), *p* < 0.0001], but there was no statistically significant difference between Tai Chi and conventional therapy in Trail-Making Test scores [MD = –3.36, 95% CI (−10.59, 3.88), *p* = 0.36] (Figures 8, 9).

**Figure 10:**
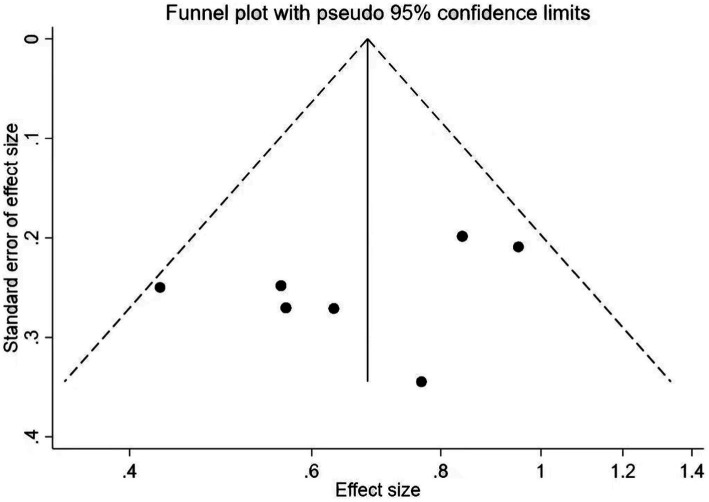
Publication bias of Trail-Making Test (TMT) scores.

The meta regression analysis of the TMT scores showed that there is no significance in intervention times [95% CI (−0.22, 0.22), *p* = 0.898], intervention duration [95% CI (−1.61, 1.57), *p* = 0.890] and single intervention time [95% CI (−0.21, 0.20), *p* = 0.887] (Table 2). And as for the control groups involving active procedures and year of publication, there is also no significance [95% CI (−4.33, 3.97), *p* = 0.869; 95% CI (−2.87, 4.03), *p* = 0.279] (Table 3). Publication bias of Trail-Making Test scores can see the funnel plot (Figure 10), Egger’s regression indicates that there is little possibility of publication bias in the included studies (*p* = 0.312).

**Table 3:** Meta-regression analysis of potential moderators TMT to explain heterogeneity of older people with cognitive impairment.

	Meta-regression coefficient	95%CI	*P*
Year of publication	0.1431971	−1.033251 to 1.319645	0.365
Intervention times	−0.0028614	−0.227172 to 0.2214491	0.898
Intervention duration	−0.021957	−1.619985 to 1.576071	0.890
Single intervention time	−0.0029838	−0.2137257 to 0.2077581	0.887
Control groups involve active procedures	0.5801148	−2.878508 to 4.038737	0.279

## Discussion

This review gathered evidence with respect to the effects of different TCEs on cognitive function in older adults with cognitive impairment and compared them with the control group. This meta-analysis showed that TCEs had acceptable beneficial results on global cognition function in elderly people with cognitive impairment, and the intervention times, intervention duration and single intervention time do not have any impact. Similarly, this review provided further insights into the additional benefits of Baduanjin in enhancing global cognition functions, memory functions, and executive functions among older adults with cognitive impairment. Therefore, the current review findings may provide referenceable evidence to apply TCEs to the older population.

The impact of TCEs, which include Baduanjin, Tai Chi, and Qigong, on the cognitive function (MoCA and MMSE) of elderly patients with cognitive impairment is statistically significant. This is consistent with the results of some studies (34, 37–39). The mechanisms by which TCEs may improve cognitive functions in individuals with MCI are still being investigated. Nevertheless, TCEs may improve cognitive functions by modifying the risk factors (such as physical inactivity, depressive symptoms and social isolation) of cognitive decline among this vulnerable population. Unlike ordinary sports, TCEs combine physical exercise with mental exercise (40). TCEs have been practiced for many centuries and is gaining popularity in the West. According to the 2007 National Health Interview Survey, 2.3 million US adults had practiced Tai Chi in the past 12 months (41).

Analysis of memory function indexes, including AVLT-short-term delayed recall, showed that TCEs improved the overall memory function of older adults with cognitive impairment, though they did not improve the scores on immediate recall and long-term delayed recall after treatment. Previous studies have suggested beneficial effects of TCEs on global cognitive function and memory in MCI or healthy older adults (42, 43). Further subgroup analysis of immediate recall showed that the curative effect of Baduanjin was better than that of conventional therapy, but there was no statistically significant difference between Tai Chi and conventional therapy in AVLT-immediate recall. Some studies recently reported that the Baduanjin was beneficial in maintaining or even improving both global cognitive function and specific domains of cognition, including memory processing speed, executive function, or verbal learning and memory in older adults with or without cognitive impairment (44–46). A study from USA indicates that Tai Chi has some impact on global cognitive functioning but not attention or memory (Hopkins Verbal Learning Test–Revised) (47). And a study from London showed that a 22 weeks Tai Chi exercise did not specifically improve cognition or memory strategy knowledge and use (48). That means, Tai Chi cannot affect the immediate recall.

The term “executive functions” is an umbrella term for a wide range of cognitive processes and behavioral competencies necessary for the cognitive control of behavior (49). Neuropsychological tests of executive functions aim to assess these processes (50). TCEs group performed significantly better in the Trail Making B-A tests than the control group. That means TCEs were effective in improving executive function in older adults with this condition. Specifically, TCEs appear to benefit the task switching component of executive function. This finding is consistent with findings from previous studies (51, 52) and suggests the potential of TCEs as an exercise regimen to improve executive function in older adults with cognitive impairment. And in our study, we found that the curative effect of Baduanjin was better than that of conventional therapy, but there was no statistically significant difference between Tai Chi and conventional therapy in Trail-Making Test scores. A meta-analysis also confirmed the same results that Tai Chi failed to support benefit for executive function in older adults (53, 54).

There is no consensus on which TCEs method should be adopted and the frequency, duration, and intensity of exercise that can maximize the benefits for the older adults. Given that in our study Baduanjin show the benefits in enhancing global cognition functions, memory functions, and executive functions among older adults with cognitive impairment. Additionally, it is a low-risk, inexpensive activity with many advantages; therefore, we suggest that Baduanjin can be implemented to prevent cognitive decline for older adults.

### Strengths and limitations

The main strengths of this study are the assessments of specific cognitive domains (cognitive, memory, and executive) often shown to be impaired at an early stage of cognitive impairment. Besides, we performed subgroup analysis on the duration time of total treatment and intensity of the TCEs varied among the global cognitive function, memory function, and executive function, which may provide insight into the factors linking TCEs to cognitive improvement.

And this systematic review and meta-analysis has several limitations. First, most of included studies were from China, the lack of other ethnic studies may lead to some bias and less convincing results. Second, none of the studies used follow-up measurements, so the long-term effects of TCEs are still difficult to predict. Lastly, the studies included in this systematic review include cognitive impairment caused by different causes, including old age, stroke, diabetes, and unspecified primary disease; it is not clear whether the beneficial effects of TCEs are applicable to cognitive impairment due to all causes. Nevertheless, our study still provides reliable information for the treatment decisions of elderly patients with cognitive impairment. In the future, we hope to obtain the best type of TCEs and effective exercise frequency, intensity, total exercise sessions, or duration time of treatment, further promote TCEs globally.

## Conclusion

In this meta-analysis, pooled analyses indicated that TCEs had considerable beneficial effects on the elderly with cognitive impairment. Analysis of multiple indexes of cognitive function after treatment showed that TCEs improved the global cognitive function of elderly people with cognitive impairment. And it is worth mentioning that Baduanjin has obvious effects on the cognitive, memory, and executive functions of the elderly with cognitive impairment.

### Clinical messages

• TCEs improved the overall cognitive function of cognitively impaired elderly people.

•Baduanjin has obvious effects on the cognitive, memory, and executive functions of the elderly with cognitive impairment.

## Data availability statement

The original contributions presented in the study are included in the article/supplementary material, further inquiries can be directed to the corresponding author.

## Author contributions

K-rY and QL: study concept and design. K-rY, QL, Z-hW, LuL, and LuZ: acquisition of data. K-rY, QL, XT, LiZ, LH, and X-hY: analysis and interpretation of data. K-rY and QL: drafting of the manuscript. K-rY, QL, LiZ, LH, LiL, and X-hY: critical revision of the manuscript for important intellectual content. K-rY, QL, LH, and X-hY: statistical analysis. K-rY and QL: study supervision. All authors contributed to the article and approved the submitted version.

## Funding

The study is supported by Postgraduate Scientific Research Innovation Project of Hunan Province (Grant No. KYCX202202), the Hunan Social Science Foundation (Grant No. XSP20ZDI007), 2019 Hunan Postgraduate Quality Course Project (Grant [2019] No. 370 248) and University of South China Project (Grant No. NK 2020209), Hunan Provincial Health Commission (Grant No. 202214015306), Hunan Provincial Department of Education (Grant No. 22C0215). The funders had no role in study design, data collection and analysis, the decision to publish, or the preparation of the manuscript.

## Conflict of interest

The authors declare that the research was conducted in the absence of any commercial or financial relationships that could be construed as a potential conflict of interest.

## Publisher’s note

All claims expressed in this article are solely those of the authors and do not necessarily represent those of their affiliated organizations, or those of the publisher, the editors and the reviewers. Any product that may be evaluated in this article, or claim that may be made by its manufacturer, is not guaranteed or endorsed by the publisher.
